# Metabolomic Profiling of Aqueous Humor and Plasma in Primary Open Angle Glaucoma Patients Points Towards Novel Diagnostic and Therapeutic Strategy

**DOI:** 10.3389/fphar.2021.621146

**Published:** 2021-04-14

**Authors:** Yizhen Tang, Yiqiong Pan, Yuhong Chen, Xiangmei Kong, Junyi Chen, Hengli Zhang, Guangxian Tang, Jihong Wu, Xinghuai Sun

**Affiliations:** ^1^ Department of Ophthalmology and Visual Science, Eye and ENT Hospital, Shanghai Medical College, Fudan University, Shanghai, China; ^2^ NHC Key Laboratory of Myopia, Chinese Academy of Medical Sciences, and Shanghai Key Laboratory of Visual Impairment and Restoration (Fudan University), Shanghai, China; ^3^ Department of Ophthalmology, Shijiazhuang No. 1 Hospital, Hebei, China; ^4^ State Key Laboratory of Medical Neurobiology and MOE Frontiers Center for Brain Science, Institutes of Brain Science, Fudan University, Shanghai, China

**Keywords:** metabolomics, biomarker, primary open angle glaucoma, plasma, aqueous humor, mass spectrometry

## Abstract

Glaucoma is the second leading cause of blindness globally characterized by progressive loss of retinal ganglion cells (RGCs) and irreversible visual deficiency. As the most common type of glaucoma, primary open angle glaucoma (POAG) is currently an unmet medical need with limited therapy by lowering intraocular pressure (IOP). However, some patients continue to progress even though their IOP are controlled. Although early diagnosis and prompt treatment are crucial in preventing irreversible visual impairment, there are currently no biomarkers for screening POAG. Metabolomics has the advantages of illustrating the final downstream products of the genome and establishing the closest link to the phenotype. So far, there is no study investigating the metabolomic profiles in both aqueous humor and plasma of POAG patients. Therefore, to explore diagnostic biomarkers, unveil underlying pathophysiology and potential therapeutic strategies, a widely targeted metabolomic approach was applied using ultrahigh-resolution mass spectrometry with C18 liquid chromatography to characterize the metabolomic profiles in both aqueous humor and plasma of 28 POAG patients and 25 controls in our study. Partial least squares-discriminant analysis (PLS-DA) was performed to determine differentially expressed metabolites (DEMs) between POAG and age-matched controls. The area under the receiver operating characteristic curve (AUC) was calculated to assess the prediction accuracy of the DEMs. The correlation of DEMs with the clinical parameters was determined by Pearson correlation, and the metabolic pathways were analyzed using MetaboAnalyst 4.0. PLS-DA significantly separated POAG from controls with 22 DEMs in the aqueous humor and 11 DEMs in the plasma. Additionally, univariate ROC analysis and correlation analysis with clinical parameters revealed cyclic AMP (AUC = 0.87), 2-methylbenzoic acid (AUC = 0.75), 3′-sialyllactose (AUC = 0.73) in the aqueous humor and N-lac-phe (AUC = 0.76) in the plasma as potential biomarkers for POAG. Moreover, the metabolic profiles pointed towards the alteration in the purine metabolism pathway. In conclusion, the study identified potential and novel biomarkers for POAG by crosslinking the metabolomic profiles in aqueous humor and plasma and correlating with the clinical parameters. These findings have important clinical implications given that no biomarkers are currently available for glaucoma in the clinic, and the study provided new insights in exploring diagnostic biomarkers and potential therapeutic strategies of POAG by targeting metabolic pathways.

## Introduction

Glaucoma is the second leading cause of blindness globally characterized by progressive loss of retinal ganglion cells (RGCs) and irreversible visual deficiency. It is predicted to affect more than 100 million people worldwide by 2040 (Tham et al., 2014). As the most common type of glaucoma, primary open angle glaucoma (POAG) is currently an unmet medical need with limited therapy by lowering intraocular pressure (IOP). However, some patients continue to progress even though their IOP are controlled. Patients with POAG are conventionally diagnosed based on several clinical and ancillary examinations. Although early diagnosis and prompt treatment are crucial in preventing permanent and irreversible visual loss of POAG (Heijl et al., 2002), there are currently no biomarkers for screening due to the complicated pathogenesis (Weinreb et al., 2014).

Although increasing methods including transcriptomics and proteomics have been utilized to unveil the complicated pathogenesis of POAG (Bhattacharya et al., 2013; Takamoto and Araie, 2014; Funke et al., 2016; Shiga et al., 2018), metabolomics has its advantages by illustrating final downstream products of the genome and establishing the closest link to the phenotype associated with the pathophysiology of diseases (Schrimpe-Rutledge et al., 2016). It is more susceptible to external perturbations, even when alterations in proteins and transcripts were not detectable (Ellis et al., 2007). All these enable metabolomics with high potential to explore novel diagnostic biomarkers for POAG disease and unveil new insights into underlying pathophysiology and potential therapeutic strategies of POAG.

To the best of our knowledge, there are only few studies investigating biomarkers in POAG *via* metabolomic approaches (Edwards et al., 2014; Burgess et al., 2015; Leruez et al., 2018; Buisset et al., 2019; Tang et al., 2019; Kouassi Nzoughet et al., 2020; Myer et al., 2020; Pan et al., 2020). Previous results showed discriminant metabolites involved in steroid biosynthesis, mitochondrial oxidation of energetic substrates, senescence and polyamines function in the plasma of POAG patients (Burgess et al., 2015; Leruez et al., 2018; Kouassi Nzoughet et al., 2020). Studies showed metabolomic profiling of aqueous humor in POAG points towards the alteration of biotin and beta-alanine metabolism (Burgess et al., 2015; Leruez et al., 2018; Kouassi Nzoughet et al., 2020), taurine and spermine (Burgess et al., 2015; Leruez et al., 2018; Kouassi Nzoughet et al., 2020) amino acid metabolism (Burgess et al., 2015; Leruez et al., 2018; Kouassi Nzoughet et al., 2020), glutamine and glutamate metabolism (Barbosa Breda et al., 2020). However, there is still no study investigating the metabolomic profiles in both aqueous humor and plasma of POAG patients to comprehensively explore the metabolic alterations.

Given the above reasons, the present study was set up to characterize the metabolomic profiles of POAG using both aqueous humor and plasma and correlate clinical parameters of individual subject with metabolomic signature to identify novel diagnostic biomarkers and explore potential therapeutic targets for POAG disease.

## Methods

### Study Participants

Individuals were recruited from the Department of Ophthalmology in two tertiary hospitals in China: Eye and ENT Hospital of Fudan University and the First Hospital of Shijiazhuang. In metabolic phenotyping, there is currently no available approach for the estimation of sample size. Therefore, the sample size in the present study was estimated based on previous studies (Lapice and Vaccaro, 2014; Pan et al., 2020). POAG was diagnosed by experienced ophthalmologists with the same criteria. The diagnostic criteria were: 1) intraocular pressure >21 mmHg, 2) glaucomatous optic nerve damage with progressive optic disc cupping (Jonas et al., 2017), 3) open iridocorneal angles determined by gonioscope examination. Patients with isolated ocular hypertension, normal tension glaucoma, secondary glaucoma, previous glaucoma surgery, other ocular diseases, and systemic diseases including diabetes, renal diseases, cardiac disease and immune diseases have been excluded from our study. No previous intraocular surgery including cataract surgery was performed in the patients recruited in our study. Laser procedure conducted at least two weeks before our study was allowed. Patients with POAG underwent ancillary tests including fundus photography, visual field testing (Humphrey field analyzer, SITA full-threshold programs 30-2; Carl Zeiss Meditec, Dublin, CA), retinal never fiber layer thickness, and ganglion cell complex evaluation using spectral-domain optical coherence tomography (Avanti RTVue-XR, Optovue, CA). The central corneal thickness, best corrected visual acuity (logMAR) were recorded for all POAG patients. The history of glaucoma treatment was also documented. Controls were sex and age-matched individuals undergoing cataract surgery at the same department of ophthalmology. The inclusion criteria for the control group were age-related cataract with no ocular conditions except for cataract. The exclusion criteria were family history of glaucoma, ocular hypertension, retinal disorders and any ocular surgery. All the patients recruited in the study were above 40 years old.

### Sample Collection

The corresponding aqueous humor (AH) and plasma of patients were collected for metabolomic analysis. Proparacaine hydrochloride was used in the operating room to prepare for surgery. Tropicamide was also used for the patients who underwent phacoemulsification and intraocular lens implantation. The AH was collected at the beginning of the surgery through a corneal paracentesis via a fine-bore needle. AH was obtained during the surgical treatment for POAG and cataract patients by experienced ophthalmologists. The surgeries that POAG patients underwent on the day of aqueous humor collection are shown in Table 1. The surgeries were performed in the morning from 8 am to 12 pm. Afterward, AH samples were transferred to sterilized cryotubes immediately and rapidly stored at −80°C for metabolomic analysis.

**TABLE 1:** Glaucoma medication and surgeries performed in the present study.

	POAG	Ctrl	
Surgery			
Trabeculectomy, %	46	0	
Drainage implant surgery, %	29	0	
Non-penetrating trabeculectomy, %	11	0	
Phacoemulsification and intraocular lens implantation, %	14	100	
Glaucoma medication			
Travoprost, %	50	-	
Brimonidine Tartrate, %	50	-	
Brinzolamide, %	46.43	-	
Brinzolamide and Timolol, %	35.71	-	
Bimatoprost, %	32.14	-	
Timolol, %	10.71	-	
Carteolol, %	10.71	-	
Mannitol, %	7.14	-	
Latanoprost, %	3.57	-	
Tafluprost, %	3.57	-	

Categorical variables were presented as proportion.

The blood was collected one day prior to the surgery when patients were admitted to the hospital. Blood samples from participants were collected in heparin tubes at least 6 h after the last meal. The tubes were immediately transported on ice and immediately processed for centrifugation for 10 min at 3,000 g at 4°C. Then the supernatant was collected and immediately stored at −80°C until metabolomic analysis.

### Metabolomics Analysis

The preparation of samples, extract analysis, metabolite identification and quantification were performed in MetWare Biotechnology Co., Ltd. following their standard procedures. Briefly, samples were thawed on ice and vortexed for 10 s to mix homogeneously. 300 μl ice-cold methanol with internal standard was added to 50 μl of the sample. The mixture was whirled and centrifuged at 16,000 g at 4°C for 10 min. The supernatant was collected in a new tube and centrifuged again at 16,000 g at 4°C for 5 min. The supernatant was then added into the liner of the corresponding injection bottle for on-board analysis. The sample extracts were analyzed using an LC-ESI-MS/MS system [Liquid Chromatography Electrospray Ionization Tandem Mass Spectrometric, Ultra Performance Liquid Chromatography (UPLC), ExionLC AD, MS, QTRAP® System]. Briefly, 2 μl volume of sample extracts was injected into Waters Acquity UPLC HSS T3 C18 (1.8 µm, 2.1 mm, and 100 mm) column. The column temperature was held constant at 40°C with a flow rate of 0.4 ml/min. The binary gradient system consisted of (A) water (0.1% formic acid) and (B) acetonitrile (0.1% formic acid) and the gradient program was as follows: 95:5 V/V at 0 min, 10:90 V/V at 11.0 min, 10:90 V/V at 12.0 min, 95:5 V/V at 12.1 min, 95:5 V/V at 14.0 min.

Linear ion trap (LIT) and triple quadrupole (QQQ) scans were acquired on a triple quadrupole-linear ion trap mass spectrometer (QTRAP), equipped with an ESI Turbo Ion-Spray interface, operating in positive and negative ion mode and controlled by Analyst 1.6.3 software (Sciex). The ESI source operation parameters were as follows: The temperature of the source was set at 500°C. The ion spray voltage was set at 5.5/−4.5 kV; ion source gas I, gas II curtain gas (CUR) were set at 55, 60, and 25.0 psi, respectively; the collision gas was high. Instrument tuning and mass calibration were performed with 10 and 100 μmol/L polypropylene glycol solutions in QQQ and LIT modes. A specific set of MRM transitions were monitored for each period according to the metabolites eluted within this period.

The quality control (QC) samples were prepared prior to sample analysis. To prevent cross-effects, the injection sequence of plasma and aqueous humor samples was separated. Both plasma and aqueous humor samples were randomized. QC samples were inserted after every 10 samples to be analyzed to monitor the repeatability during the analysis process. The high overlaps of the total ion chromatography (TIC) and high coefficient of variate (CV) quality between QC samples indicate good signal stability of the mass spectrum at different time. Standards were used to confirm the identity of metabolites based on the self-built target standard database MWDB (metware database) by widely target UPLC-MS/MS platform of Metware Biotechnology Co., Ltd. Qualitative analysis was carried out according to the retention time (RT), the information of the precursor ion pair and the secondary spectrum data of the detected metabolites. Metabolite quantification was accomplished by using multiple reaction monitoring (MRM) analysis via triple quadrupole mass spectrometry. We used the metabolites Q1 (parent ions), Q3 (fragment) and RT under the MRM mode to identify the metabolites based on the database containing 1,200 metabolites. The metabolites with area > 1,000, signal-to-noise ratio > 3 and CV < 50% were finally identified in each sample. After the selection process, a total of 395 metabolites were recorded. Analysis of mass spectrometric data was conducted based on the Metware database (MWDB) and the analysis of raw mass spectrometry data was processed using Analyst 1.6.3 software (AB Sciex).

### Data Processing and Statistical Analysis

Univariate analysis of clinical quantitative data was performed with parametric test (Welch’s t-test) or nonparametric test (Wilcoxon-Mann Whitney test) according to the normality assessed by Shapiro Wilk’s test. Analysis of clinical qualitative gender data was performed with Fisher's exact test. *p* < 0.05 was considered statistically significant.

For univariate analysis of metabolomic data, Shapiro Wilk's test was performed to assess each variable’s normality and decide whether a parametric test (Welch’s t‐test) or a nonparametric test (Wilcoxon‐Mann Whitney test) should be performed. The analysis showed a merged set of *p*‐values from both tests, and then a multiple testing correction (Benjamini-Hochberg FDR, taking FDR < 0.05 as a threshold) was conducted to get adjust *p* value (*q*-value). Multivariate model Partial least squares-discriminant analyses (PLS-DA) were used to determine the differentially expressed metabolites in the study. The data were processed by log transformation and Pareto scaling, which stays closer to the original measurement and sensitive to large fold changes (van den Berg et al., 2006). Variable importance in projection score (VIPs) > 1, fold changes (FC) > 1.5, and q values < 0.05 were used to determine the differentially expressed metabolites (DEMs). Pathways enrichment analysis of DEMs was conducted using MetaboAnalyst v4.0 and KEGG database (Chong et al., 2018). The receiver operating characteristic (ROC) curves in logistic regression with leave one out cross-validation were used for DEMs. Pearson correlation was performed between the expression of clinical parameters and DEMs in the POAG group. A multivariate linear regression model was built for each DEM in POAG group to assess the influence of the preoperative topical drugs. F statistics were calculated for the overall medication effect, and t statistics were performed for individual medication. *p* < 0.05 was considered statistically significant.

## Result

### Subjects Characteristics

Metabolomics analysis was performed on 53 subjects, 28 subjects are POAG and 25 subjects are age-matched cataract controls. The demographic and basic clinical information of the two groups were shown in Table 2. No statistically significant differences were observed for gender and age between the two groups. And there was no difference regarding anterior chamber depth (ACD), body mass index (BMI) and central corneal thickness (CCT) in POAG and controls. Meanwhile, intraocular pressure (IOP), cup/disc ratio (C/D), best-corrected visual acuity (BCVA) and axial length (AL) were significantly higher in POAG patients, as shown in Table 2. The retinal nerve fiber layer thickness (RNFL), ganglion cell complex (GCC) parameters in the POAG group were documented in Supplementary Table S1.

**TABLE 2:** Demographic data of enrolled POAG and control subjects.

	POAG	Ctrl	*p* value
Age (y)	58.89 ± 14.9	65.60 ± 11.32	0.0735^a^
Female (%)	43	58	0.404^b^
C/D	0.9 (0.8, 0.9)	0.3 (0.3, 0.4)	<0.001^c^
IOP (mmHg)	22.97 ± 9.43	15.10 ± 2.13	<0.001^a^
BCVA (logMAR)	0.55 ± 0.45	1.2 ± 0.55	0.026^a^
BMI (kg/m^2^)	23.64 ± 2.08	25.12 ± 4.96	0.47^a^
ACD (mm)	2.7 ± 0.39	3.06 ± 0.67	0.37^a^
AL (mm)	24.27 ± 1.42	23.42 ± 0.64	0.041^a^
CCT (µm)	525.96 ± 30.13	534.00 ± 24.04	0.34^a^
MD	19 ± 8.4	—	—

AL, axial length; ACD, anterior chamber depth; BMI, body mass index; BCVA, best corrected visual acuity; C/D, cup/disc ratio; CCT, central corneal thickness; IOP, intraocular pressure; MD, main defect. Continuous variables were presented as mean ± SD or median (lower quartile - upper quartile) according to the normality of the data. Categorical variables were presented as proportion. Statistical test:

^a^ Welch’s t–test.

^b^ Fisher's exact test.

^c^ Wilcoxon–Mann Whitney test.

### Metabolomic Profile and Potential Biomarkers in Aqueous Humor

**FIGURE 1:**
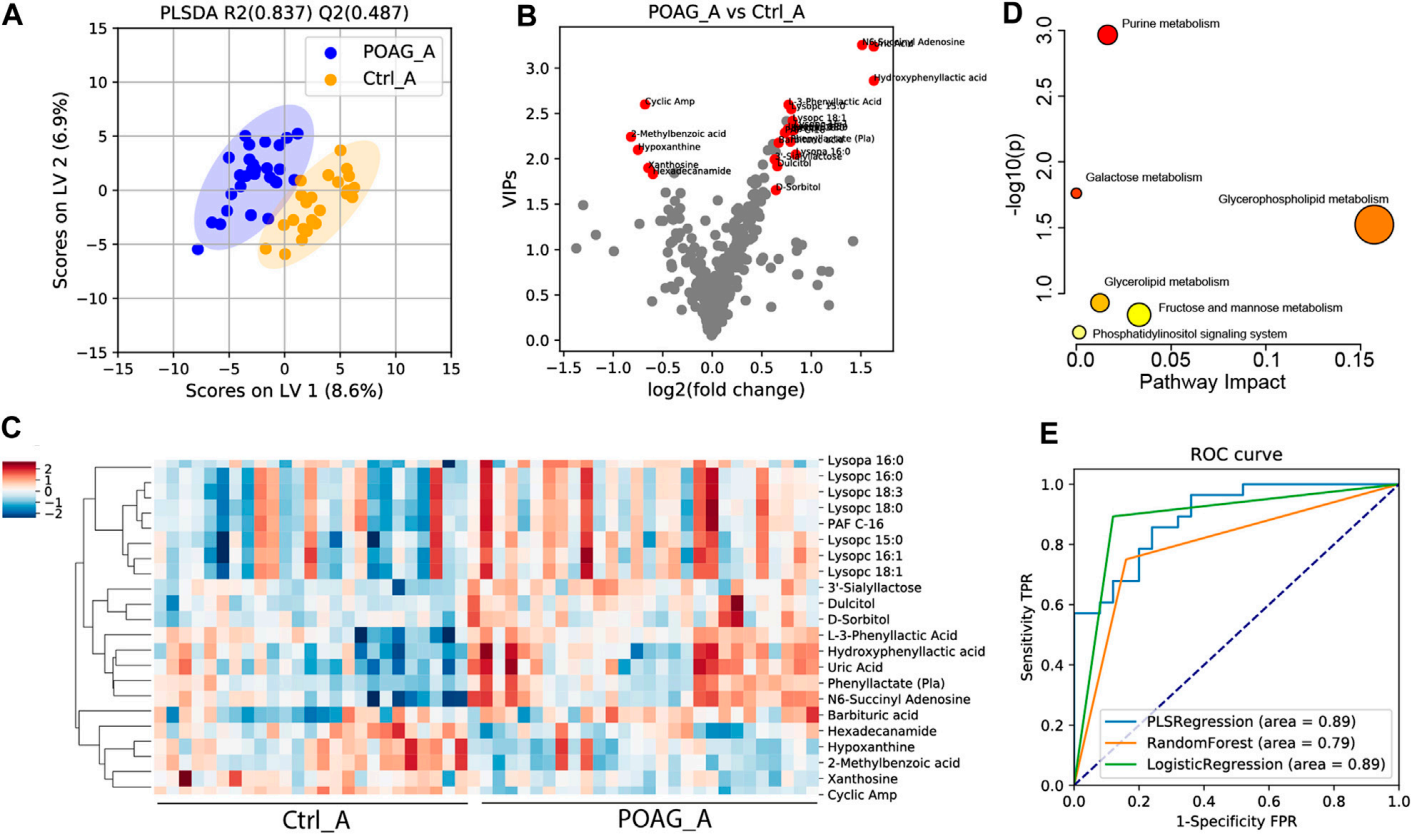
Metabolomic profile in the aqueous humor of POAG and control subjects. **(A)** PLS-DA score plot of the metabolites. **(B)**Volcano plot of variable importance projection scores (VIP). Red points labeled the differentially expressed metabolites (DEMs) with VIPs >1, q < 0.05 and FC > 1.5. **(C)** Heatmap of DEMs in the aqueous humor between POAG and control. **(D)** Pathway enrichment analysis of DEMs in KEGG metabolites set. **(E)** ROC curve analysis of DEMs using PLS regression, random forest and logistic regression models.

To determine the discriminatory metabolomic profiles between POAG and control groups in the aqueous humor, a supervised PLS-DA model was applied (Mendez et al., 2019; Kouassi Nzoughet et al., 2020; Figure 1A). The PLS-DA model explained most of the variances between groups (R^2^: 0.837) and distinguished each other with cross-validation prediction quality [Q^2^: 0.487, which is acceptable for biological models (Worley and Powers, 2013)]. Based on PLS-DA results, the variable importance in projection score (VIPs), fold changes (FC) and q values were calculated, and VIP >1, FC > 1.5 and q < 0.05 were considered differentially expressed metabolites (DEMs) in our study. The score-plot was shown in Figure 1B. 22 DEMs in aqueous humor (DEMs-A) were identified. 5 DEMs-A [cyclic AMP (cAMP); 2-methylbenzoic acid; hypoxanthine; xanthosine; hexadecanamide] were decreased and 17 DEMs-A [3′-sialyllactose; lysopc 18:0; dulcitol; lysopc 15:0; uric acid; phenyl lactate (Pla); lysopc 16:0; lysopc 18:3; Hydroxyphenyl lactic acid; lysopa 16:0; lysopc 16:1; barbituric acid; L-3-phenyllactic acid; PAF C-16; N6-succinyl adenosine; lysopc 18:1; D-sorbitol] were increased in POAG group. The distinguished expression pattern of the DEMs-A was shown in the heatmap (Figure 1C) and the DEMs-A were enriched in purine metabolism, galactose metabolism and glycerophospholipid metabolism (*p* < 0.05, Figure 1D).

To assess the diagnostic capability of DEMs-A as potential biomarkers, ROC analysis was performed. Combining all the 22 DEMs-A, the AUC score was 0.89, 0.79, and 0.89 using PLS regression, logistic regression, random forest models (Couronné et al., 2018; Mendez et al., 2019) respectively (Figure 1E). Meanwhile, the AUC score for each DEM-A was also calculated by univariate ROC analysis (Table 3). Metabolites with AUC > 0.7 were reported to contribute to the forecast classification for the individual subject among groups (Mandrekar, 2010). Our results showed that the top 3 metabolites based on the AUC scores were cAMP (AUC: 0.87), 2-methylbenzoic acid (AUC: 0.75) and 3′-sialyllactose (AUC: 0.73), indicating their high prediction capability for POAG.

**TABLE 3:** Statistics, AUC scores and clinical relevance for DEMs in the aqueous humor.

Metabolites	VIP	log_2_ (FC)	*q* value	AUC	Clinical relevance
Cyclic Amp	2.60	−0.68	<0.001	0.87	9
2-Methylbenzoic acid	2.24	−0.82	0.006	0.75	3
3’-Sialyllactose	1.99	0.63	0.006	0.73	4
Lysopc 18:0	2.32	0.82	0.03	0.71	0
Dulcitol	1.92	0.66	0.006	0.70	2
Lysopc 15:0	2.55	0.80	0.031	0.70	0
Hypoxanthine	2.10	−0.75	0.006	0.69	0
Uric Acid	3.24	1.63	0.009	0.68	0
Phenyllactate (Pla)	2.19	0.79	0.006	0.68	1
Xanthosine	1.90	−0.65	0.03	0.66	9
Lysopc 16:0	2.31	0.79	0.025	0.66	0
Lysopc 18:3	2.32	0.76	0.03	0.66	0
Hydroxyphenyllactic acid	2.86	1.63	0.013	0.66	0
Lysopa 16:0	2.05	0.84	0.03	0.66	0
Lysopc 16:1	2.35	0.83	0.041	0.66	1
Barbituric acid	2.18	0.67	0.04	0.65	1
L-3-Phenyllactic Acid	2.60	0.77	0.013	0.64	0
PAF C-16	2.29	0.73	0.037	0.60	0
N6-Succinyl Adenosine	3.25	1.51	0.012	0.60	0
Hexadecanamide	1.83	−0.60	0.038	0.60	2
Lysopc 18:1	2.42	0.81	0.034	0.58	0
D-Sorbitol	1.65	0.64	0.037	0.58	1

Clinical relevance: number of correlated clinical parameters in C/D; IOP; BCVA; AL; CCT; ACD; C/D A-Ratio; C/D V-Ratio; C/D H-Ratio; Rim Area; Disc Area; Cup Volume; GCC average thickness, GCC superior thickness; GCC inferior thickness; GCC-FLV; GCC-GLV; RNFL average thickness; RNFL superior thickness; RNFL inferior thickness and MD.

To further evaluate the correlation between the DEMs-A with clinical parameters in POAG subjects, the Pearson correlation was performed (Supplementary Table S2). The results revealed 4 potential biomarkers with significant correlation (*p* < 0.05) of more than 3 clinical parameters. cAMP was shown to be correlated with 9 clinical parameters (Table 3) including positive correlation with rim area, GCC average thickness, GCC superior thickness, GCC inferior thickness, RNFL average thickness, RNFL superior thickness, RNFL inferior thickness, and negative correlation with C/D and GCC-GLV. Xanthosine was also correlated with 9 clinical parameters including positive correlation with rim area, disk area, GCC superior thickness, RNFL average thickness, RNFL superior thickness, RNFL inferior thickness, and negative correlation with C/D, C/D V-ratio, and GCC-GLV. 3′-sialyllactose was correlated with 4 clinical parameters including positive correlation with C/D A-ratio, C/D V-ratio, C/D H-ratio, and negative correlation with RNFL superior thickness. 2-methylbenzoic acid was correlated with 3 clinical parameters including positive correlation with GCC average thickness and negative correlation with C/D H-ratio and cup volume. All the data reflected the significant clinical relevance of the selected metabolites.

To assess the influence of the preoperative topical drugs on the metabolites, a multivariate linear regression model was built for each DEMs-A in the POAG group. The results showed that the drugs have significant impact on uric acid, Pla and N6-succinyl adenosine (Supplementary Table S6). To evaluate the influence of the administration of tropicamide before cataract surgery for DEMs in the aqueous humor, a multivariate linear regression analysis was performed and the results showed only lysopa 16:0 (*p* < 0.05) was affected by tropicamide (Supplementary Table S5).

**FIGURE 2:**
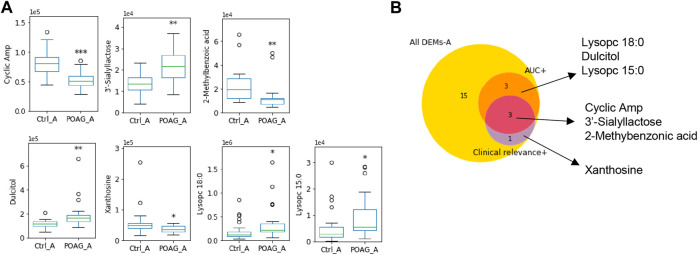
Metabolomic biomarkers in the aqueous humor of POAG patients. **(A)** The expression of the metabolites biomarkers including cAMP, 3′-sialyllactose, 2-methylbenzoic acid, Dulcitol, xanthosine, lysopc 18:0 and lysopc 15:0. **(B)** Venn map of biomarker selection criteria (AUC > 0.7, clinical relevance ≥3) for DEMs-A. ***: *p* < 0.001, **: *p* < 0.01, *: *p* < 0.05 by unpaired t-test compared to controls. Data were represented in box-and-whisker plot.

Taken the results of ROC analysis and correlation analysis with clinical parameters together, cAMP, xanthosine and 3’-sialyllactose stood out with AUC scores above 0.7 and clinical relevance with more than 3 parameters (Table 3). The expression level of these biomarkers and their selection criteria were summarized in Figure 2.

### Metabolomic Profile and Potential Biomarkers in Plasma

The same analyses were applied to plasma samples to determine the differentially expressed metabolites between POAG and controls. A supervised PLS-DA model was applied to characterize the differences between the two groups (Figure 2A). Based on the PLS-DA (Q^2^: 0.464, R^2^: 0.815) results, 11 DEMs in plasma (DEMs-P) were identified (Figure 2B), 5 DEMs-P (D-mannitol; inosine; hypoxanthine; hypoxanthine-9-β-d-arabinofuranoside and P-aminobenzoate) were increased and 6 DEMs-P (3-propionic acid; N-lactoyl-phenylalanine (N-lac-phe); 9-hpode; guanidinoethyl sulfonate; hydroxyacetone; and 2-aminoadipic acid;) were decreased in POAG group. The heatmap of DEMs-P and the enrichment analysis were shown in Figures 2C,D. Purine metabolism pathway was highly enriched and lysine degradation were highly impacted.

ROC analysis was further performed to estimate the predictability of DEMs-P for potential biomarkers. Combining all the 11 DEMs-P, the AUC scores were 0.89, 0.79, 0.76 (for PLS regression, logistic regression, random forest model, respectively) (Figure 2E). The AUC score for each DEMs-P was also calculated by univariate ROC analysis (Table 4) and the top 3 metabolites were 3-propionic acid (AUC: 0.82), N-lac-phe (AUC: 0.76) and 9-hpode (AUC: 0.74), indicating their high diagnostic capability as biomarkers for POAG.

**TABLE 4:** Statistics, AUC scores and clinical relevance for DEMs in the plasma.

Metabolites	VIP	log_2_ (FC)	*q* value	AUC	Clinical relevance
3-(4-Hydroxyphenyl)-Propionic Acid	1.87	−0.79	0.047	0.82	0
N-lactoyl-phenylalanine	1.61	−0.65	0.04	0.76	2
9-Hpode	2.42	−1.41	0.047	0.74	0
D-Mannitol	2.61	1.50	0.04	0.71	0
Inosine	3.18	5.72	0.04	0.70	1
Hypoxanthine	2.03	2.04	0.04	0.66	0
Guanidinoethyl Sulfonate	1.83	−0.60	0.048	0.65	1
Hypoxanthine-9-β-D-Arabinofuranoside	2.98	5.14	0.04	0.65	1
P-Aminobenzoate	1.87	2.03	0.047	0.63	0
Hydroxyacetone	2.02	−1.00	0.047	0.60	3
2-Aminoadipic Acid	1.42	−0.73	0.048	0.57	2

Clinical relevance: number of the correlated clinical parameters in C/D; IOP; BCVA; AL; CCT; ACD; C/D A-Ratio; C/D V-Ratio; C/D H-Ratio; Rim Area; Disc Area; Cup Volume; GCC average thickness, GCC superior thickness; GCC inferior thickness; GCC-FLV; GCC-GLV; RNFL average thickness; RNFL superior thickness; RNFL inferior thickness and MD.

Pearson correlation was performed to evaluate the correlation between the DEMs-P with clinical parameters in POAG subjects (Supplementary Table S3; Table 4). Hydroxyacetone was in negative correlation with C/D, IOP and CCT. N-lac-phe was in negative correlation with CCT and disk area. 2-aminoadipic acid was in negative correlation with C/D and IOP. And no significant drug effect was found for DEMs-P (Supplementary Table S6).

Taken together, N-lac-phe stood out with an AUC score of 0.76 and clinical relevance with 2 parameters, which may serve as a potential biomarker for POAG patients in the plasma (Table 4). The expression level of these biomarkers and their selection criteria were summarized in Figure 4.

### Comparison Between Aqueous Humor and Plasma

To crosslink the metabolic profiles in plasma and aqueous humor of POAG patients, the correlation of each metabolite between aqueous humor and plasma was analyzed using Pearson correlation. Among the total 395 metabolites, 117 (approximately 30%) were significantly correlated (*p* < 0.05) in aqueous humor and plasma. 108 of the 117 showed a positive correlation, and 9 showed a negative correlation. Interestingly, 5 DEMs out of the 33 DEMs were found significantly correlated (*p* < 0.05) between aqueous humor and plasma, which also occupied approximately 15% (Table 5). Four of them (barbituric acid, lysopa 16:0, N-lac-phe and cAMP) showed a positive correlation while N6-succinyl adenosine showed a negative correlation (r = −0.47, *p* < 0.05).

**TABLE 5:** The correlation of DEMs between aqueous humor and plasma.

Metabolites	Correlation^a^		Aqueous humor^b^			Plasma^b^		
	r	*p* value	VIP	log_2_ (FC)	*p* value	VIP	log_2_ (FC)	*p* value
Barbituric acid	0.84	<0.001	2.2	0.67	0.005	0.77	−0.074	0.46
Lysopa 16:0	0.58	0.005	2	0.84	0.003	0.76	0.062	0.36
N6-succinyl adenosine	−0.47	0.028	3.3	1.5	<0.001	1.7	−0.41	0.006
N-lactoyl-phenylalanine	0.46	0.03	0.67	−0.033	0.8	1.6	−0.65	0.001
Cyclic AMP	0.42	0.05	2.6	−0.68	<0.001	1.3	−0.37	0.013

^a^ The correlation of each metabolite between aqueous humor and plasma was analyzed using Pearson correlation.

^b^ Statistics of the metabolites in POAG group compared to control group.

## Discussion

In this paper, a widely targeted metabolomic approach was applied to explore potential diagnostic biomarkers and possible therapeutic targets of POAG and control subjects in both aqueous humor and plasma. A total of 33 DEMs were identified capable of discriminating POAG from controls. Further, AUC analysis and correlation analysis with clinical parameters were performed to explore potential diagnostic biomarkers for POAG disease. Taken together, cAMP, 2-methylbenzoic acid, 3′-sialyllactose in the aqueous humor and N-lac-phe in the plasma were identified as candidates for biomarkers of POAG based on high AUC score and clinical relevance. Moreover, the enrichment analysis of the DEMs pointed towards the alteration in purine metabolism.

### Biomarkers in Aqueous Humor for POAG

Based on the results, cAMP in the aqueous humor has the highest diagnostic potential for POAG disease. cAMP significantly decreased in both aqueous humor [*p* < 0.001, log_2_ (FC) = −0.68] and plasma [*p* < 0.05, log_2_ (FC) = −0.37], and the concentration was significantly correlated (r = 0.42, *p* = 0.05) between aqueous humor and plasma. cAMP regulates numerous biological processes in the central nervous system including the retina and optic nerve (Shim et al., 2017). A recent study reviewed that cAMP generated by adenylyl cyclases (ACs) was important not only in regulating aqueous humor dynamics and IOP regulation (Majeed et al., 2015), but also in retina ganglion cell (RGC) survival (Goldberg et al., 2002; Russo et al., 2015; Shim et al., 2017) and microglial activation (Ghosh et al., 2012). ACs, as the enzymes that catalyze the synthesis of cAMP from adenosine 5′-triphosphate (ATP), could thus be the potential drug targets in neurodegenerative disease including glaucoma. Moreover, cAMP can also be modulated by protein kinase A (PKA), which is critical in regulating glycogen, sugar and lipid metabolism. Targeting the cAMP-PKA signaling pathway is thus promising for protecting RGC (Zhou et al., 2019). Collectively, locally targeting the generation of cAMP or activation of ACs could be the potential therapeutic strategy and further investigations are needed.

2-Methylbenzoic acid is also a promising diagnostic biomarker in the aqueous humor identified in our study. It is the degradation product of xylene and benzoate. Xylene is toxic to human health by causing visual impairment and nervous system abnormality via gamma-aminobutyric acid (GABA) (Niaz et al., 2015). Benzoate/benzoic acid is the byproduct of phenylalanine metabolism through β oxidation (Widhalm and Dudareva, 2015) and it is often conjugated to glycine in the liver and excreted as hippuric acid. Benzoic acid is reported to reduce inflammation and suppressing the activation of microglia (Brahmachari et al., 2009; Jana et al., 2013). Currently, the effect of benzoic acid on glaucomatous disease is largely unknown. In our study, the concentration of both benzoic acid [*p* = 0.02, log_2_ (FC) = −0.5] and its derivates metabolites like 2-methylbenzoic acid [*p* < 0.01, log_2_ (FC) = −0.82] and p-aminobenzoate [*p* = 0.01, log_2_ (FC) = −0.47] were decreased in the aqueous humor of POAG patients, indicating its involvement in POAG pathology.

3′-sialyllactose was significantly increased in the aqueous humor of POAG patients in our study. It is reported to be anti‐inflammatory and contribute to immune homeostasis (Donovan and Comstock, 2016). 3′-sialyllactose affected the microbiota by supporting the growth of beneficial gut bacteria (Gourbeyre et al., 2011). It is also crucial for brain development and cognitive functions (Wang and Brand-Miller, 2003) and administration of 3′-sialyllactose diminish anxiety-like behavior in mice (Tarr et al., 2015). The effect of 3′-sialyllactose on glaucomatous disease is largely unknown and needs to be elucidated.

### Biomarkers in Plasma for POAG

N-lac-phe came out as a potential diagnostic biomarker with an AUC score of 0.76 and decent correlation relevance with CCT and disk area in the present study. N-lactoyl-phenylalanine (N-lac-phe) is a lactoyl derivative of phenylalanine that is involved in the formation of neurotransmitters and the pathophysiology of neurodegeneration (Socha et al., 2019). L-phenylalanine has been reported decreased in the aqueous humor (Myer et al., 2020) and tears (Rossi et al., 2019) of POAG patient. In our study, both N-lac-phe, its originative L-phenylalanine [*p* = 0.02, log_2_ (FC) = −0.22] and its oxidation product phenyllactate [*p* = 0.01, log_2_ (FC) = −0.47] were significantly decreased in POAG plasma, revealing their involvements in POAG pathology. Moreover, 9-hpode was metabolized from linoleic acid via cytochrome P450 microsomal enzymes (Ayala et al., 2014). Our results showed that 9-hpode was decreased [*p* < 0.01, log_2_ (FC) = −1.4] while linoleic acid (C18:2N6C) was not changed [*p* = 0.76, log_2_ (FC) = −0.05] in the plasma of POAG patients. Interestingly, this is in accordance with the fact that POAG patients suffered the dysfunction of cytochrome P450 (Banerjee et al., 2016) and the dysregulation in mitochondrial function. These were in line with previous reports that mitochondrial oxidation decreased in POAG disease (Abu-Amero et al., 2006; Leruez et al., 2018).

From the 11 DEM in plasma found in our study, some of them might be potentially related to patients' diet. 9-hpode and hydroxyacetone are the byproducts of polyunsaturated fatty acids and 2-aminoadipic acid was considered as a potential biomarker for obesity. They were found decreased in POAG patients, which might due to the different diet. Decreased 3-(4-hydroxyphenyl)-propionic acid and phenylalanine in POAG patients were also found related to the diet. A study has reported that 3‐(3‐hydroxyphenyl) propionic acid formed by human microflora decreases arterial blood pressure in rats (Najmanová et al., 2016). Short-term intake of red wine and grape juice extract has been shown to increase the level of 3-hydroxyphenylpropionic acid in urine and plasma and this diet is considered to promote cardiovascular health (Jacobs et al., 2012). Phenylalanine is an essential amino acid that must be obtained through diet since the human body is unable to produce enough phenylalanine on its own. Phenylalanine is consumed either through food or supplementation, including wheat germ, oats, milk products, and meats. It was also recommended that the administration of D-phenylalanine might attenuate the symptoms of Parkinson’s disease (Hirayama et al., 2016).

In the present study, the enrichment analysis of the DEMs pointed towards purine metabolism, the disorder of which generally indicates dysfunction in energy production and imbalanced neuromodulation (Fumagalli et al., 2017). Moreover, cAMP and xanthosine in the aqueous humor, and xanthine in the plasma were highly clinical-relevant, implying the critical involvement of purine metabolism in the pathology of the POAG disease. As the building blocks for nucleic acid synthesis, purine plays a vital role in many essential biochemical processes and metabolic signals (Fumagalli et al., 2017). Disorder of purine metabolism has been linked to alteration in synaptic transmission, glia function, and neuronal plasticity (Micheli et al., 2011; Jinnah et al., 2013; Fumagalli et al., 2017). In our study, total of 12 metabolites in purine metabolism were measured (Supplementary Table S4). Distinguished expression patterns were found in the aqueous humor and plasma. In the former, most intermediate metabolites were decreased while uric acid was increased. On the contrary, most intermediate metabolites including adenosine, inosine, hypoxanthine and guanosine were increased while uric acid was not changed in the plasma of the POAG patients. Xanthine oxidoreductase (XOR) catalyzes the oxidation of hypoxanthine to xanthine and further catalyzes the oxidation of xanthine to uric acid. It represented the most relevant pathway involved in uric acid overproduction (Maiuolo et al., 2016), which might be impaired in POAG patients. What's more, uric acid, as the end product of purine metabolism in human, is one of the most important antioxidants in human biological fluids mitigating oxidative damage (Ames et al., 1981). Increasing evidence indicated that higher plasma uric acid levels might have a protective effect on CNS diseases and other life‐shortening disorders (Lolekha et al., 2015; Vieru et al., 2016). A recent study demonstrated that decreased uric acid levels might be associated with an increased risk of POAG (Li et al., 2019). In line with previous studies, our results showed decreased uric acid in the plasma of POAG patients. However, uric acid in the aqueous humor was found increased. More investigations in the future need to be carried out.

To answer the question that whether the distinct metabolomic profile of POAG in the aqueous humor and plasma was due to topical treatments, we analyzed the differences in metabolomic profile of aqueous humor and plasma in POAG patients and found 102 differential expressed metabolites (q < 0.05, FC > 1.5, VIP > 1). A multivariate linear regression model was performed to assess the influence of the preoperative topical drugs on the metabolites, and the results showed that 9 of 102 metabolites (including arachidic acid (C20:0), hexadecanedioic acid, lysope 18:2 (2N Isomer), N-acetyl-L-histidine, cis-gondoic acid, lysoPE (16:1 (9Z)/0:0), indole-3-carboxaldehyde, (±)12-HETE, 2-aminoethanesulfinic acid) were potentially affected by drugs. However, the drug effect is relatively small. Moreover, the PCA results of all the samples (Suplementary Figure S1) showed apparent separation between aqueous humor and plasma in both control and POAG groups, indicating a significant difference in metabolomic profile in aqueous humor and plasma.

**FIGURE 3:**
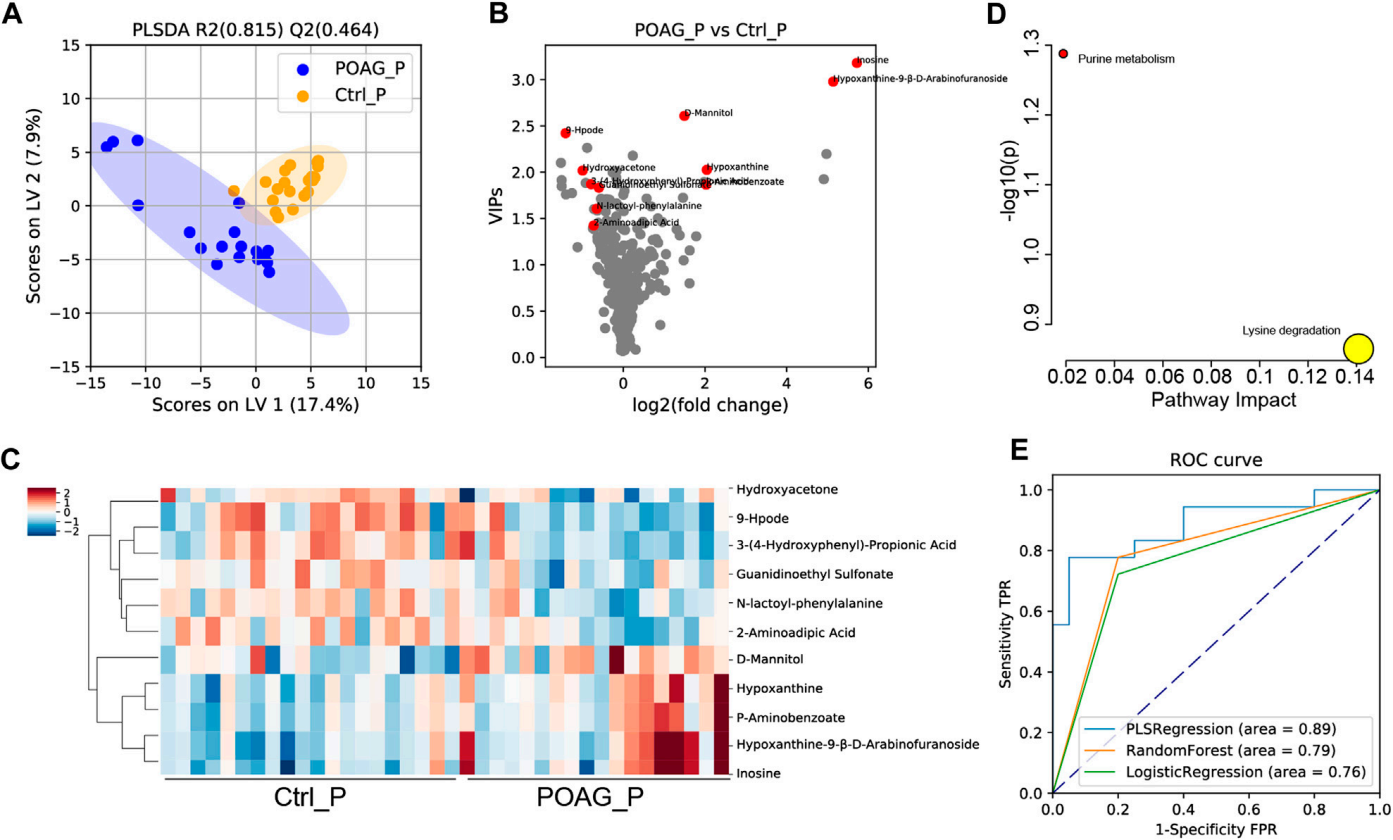
Metabolomic profile in the plasma of POAG and control subjects. **(A)** PLS-DA score plot of the metabolites in POAG and control group. **(B)**Volcano plot of VIPs. Red points labeled the DEMs with VIPs >1, q < 0.05 and FC > 1.5. **(C)** Heatmap of DEMs in the plasma between POAG and control group. **(D)** Pathway enrichment analysis of DEMs in KEGG metabolites set. **(E)** ROC curve analysis of DEMs with PLS regression, random forest and logistic regression models.

In summary, the metabolomic profile of POAG in the aqueous humor is distinct from that in plasma. It was reported that aqueous humor samples are more sensitive to detect biomarkers in glaucomatous eyes (Lolekha et al., 2015; Vieru et al., 2016). Based on our results, the AUC scores of DEMs from aqueous humor are higher than those from plasma (Figures 1E; Figure 3E). What’s more, DEMs from aqueous humor have higher clinical relevance (Tables 3 Tables 4). However, aqueous humor metabolomic profile might be influenced by topical drugs, and plasma metabolites are essential to identify biological dysfunctions in a different viewpoint and provide thorough information on metabolic alteration in POAG patients. We realize that there are some limitations in our study as the sample size is relatively small in some geographical regions, which may not be generalized to all species. Further investigations with a larger sample size are needed. One patient in our study had laser treatment two weeks prior to aqueous humor collection, which might introduce a bias. Besides, the topical eye drops of POAG patients could affect the metabolomic profile in the aqueous humor and the metabolites in plasma may be affected by patients’ diet. Moreover, the different surgeries patients underwent and the different drops given before surgery might result in another source of bias in the aqueous humor. Although the aqueous humor was collected within a timeframe of 8 am–12 pm, we should consider the difference of time might affect the metabolomic profile. Also, we need to consider the effect of cataract-related bias since we used age-related cataract patients as controls. Nevertheless, this is an inherent limitation as aqueous humor collection from healthy individuals would violate ethics. The present study provided novel diagnostic biomarkers for POAG by crosslinking the metabolic profiles in both aqueous humor and plasma and correlating with the clinical parameters. We also demonstrated that the metabolic profiles of POAG patients point towards the alteration in purine metabolism pathway. These findings have significant clinical implications for glaucoma by uncovering new insights in exploring diagnostic biomarkers and potential therapeutic strategies for POAG.

**FIGURE 4:**
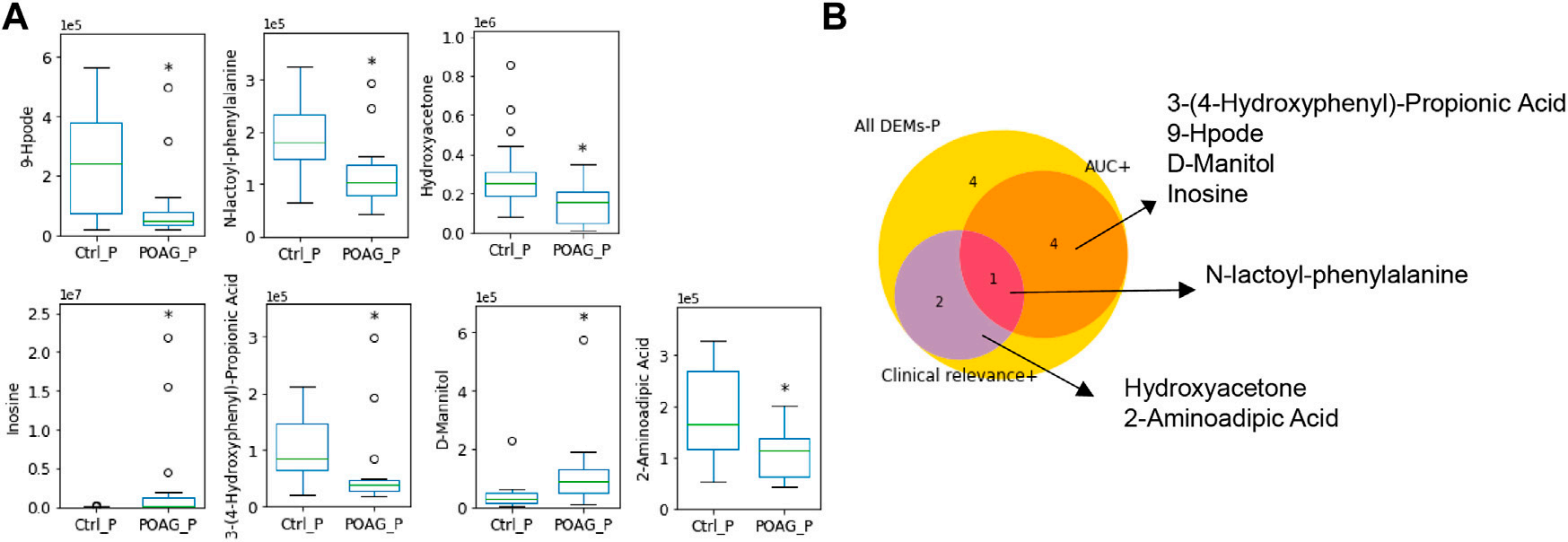
Metabolic biomarkers in the plasma of POAG patients. **(A)** The expression of the metabolites biomarkers including 9-hpode, N-lactoyl-phenylalanine, hydroxyacetone, inosine, 3-(4-hydroxyphenyl)-propionic acid, D-mannitol and 2-aminoadipic acid. **(B)** Venn map of biomarker selection criteria (AUC > 0.7, clinical relevance ≥2) for DEMs in the plasma. **: *p* < 0.01, *: *p* < 0.05 by unpaired t-test compared to controls. Data were represented in box-and-whisker plot.

## Data Availability

The original contributions presented in the study are included in the article/Supplementary Material, further inquiries can be directed to the corresponding author

## Abbreviations

AH, aqueous humor; AL, axial length; ACD, anterior chamber depth; AUC, area under the receiver operating characteristic curve; BMI, body mass index; BCVA, best corrected visual acuity; C/D, cup/disc ratio; C/D A-ratio, C/D area ratio; C/D V-ratio, C/D vertical ratio; C/D H-ratio, C/D horizontal ratio; CCT, central corneal thickness; DEMs, differentially expressed metabolites; GCC, ganglion cell complex; GCC-FLV, GCC-focal loss volume; GCC-GLV, GCC-global loss volume; IOP, intraocular pressure; LC-ESI-MS/MS, liquid chromatography electrospray ionization tandem mass spectrometric; MD, main defect; POAG, primary open-angle glaucoma; PLS-DA, partial least squares-discriminant analyses; ROC, receiver operating characteristic; RNFL, retinal nerve fiber layer thickness
