# Supplementary material for: Metabolomic Profiling of Aqueous Humor and Plasma in Primary Open Angle Glaucoma Patients Points Towards Novel Diagnostic and Therapeutic Strategy
Source: Front Pharmacol. 2021 Apr 14;12:621146. doi: 10.3389/fphar.2021.621146 (PMC8080440; doi:10.3389/fphar.2021.621146)
Supplement: Supplementary file 1 [file datasheet1.docx]

Supplementary Material

# Supplementary Figures and Tables

1. Table S1. Clinical characters of enrolled POAG patients.
2. Table S2. Details of clinical correlation for DEMs in the aqueous humor of POAG patients.
3. Table S3. Details of clinical correlation for DEMs in the plasma of POAG patients.
4. Table S4. Purine metabolites correlation between aqueous humor and plasma.
5. Table S5. The influence of the administration of tropicamide for DEMs in the aqueous humor.
6. Table S6. The overall drugs influence of the preoperative topical drugs on DEMs in POAG group.
7. Figure S1. The PCA score plots of all samples.
8. Figure S2. The correlation matrix heat map of DEMs.
9. Figure S3. Metabolite quantification process.
10. Figure S4. Quality control.
11. Figure S5. TIC_overlap chromatography data of aqueous humor samples.
12. Figure S6. Venn diagram of metabolite numbers in groups.
13. Figure S7. OPLS-DA result of aqueous humor sample.
14. Figure S8. OPLS-DA result of plasma sample.

Table S1. Clinical characters of enrolled POAG patients

|  | POAG | |
| --- | --- | --- |
|  | mean | std |
| C/D | 0.86 | 0.1 |
| IOP | 22.97 | 9.43 |
| BCVA | 0.55 | 0.45 |
| ACD | 2.70 | 0.39 |
| CCT | 525.96 | 30.13 |
| AL | 24.27 | 1.4 |
| MD | 19 | 8.4 |
| C/D A-ratio | 0.72 | 0.17 |
| C/D V-ratio | 0.86 | 0.12 |
| C/D H-ratio | 0.88 | 0.14 |
| Rim area | 0.57 | 0.23 |
| Disc area | 2.17 | 0.57 |
| Cup volume | 0.63 | 0.4 |
| GCC average | 78.67 | 22.59 |
| GCC superior | 78 | 14.63 |
| GCC inferior | 79.58 | 34.04 |
| GCC-FLV | 10 | 5.70 |
| GCC-GLV | 23.76 | 11.44 |
| RNFL average | 68.5 | 11.00 |
| RNFL superior | 73.67 | 11.88 |
| RNFL inferior | 63.41 | 11.02 |

Variables presented as mean ± standard deviation. AL: axial length; ACD: anterior chamber depth; BCVA: best corrected visual acuity; C/D: cup/disc ratio; C/D A-ratio: C/D area ratio; C/D V-ratio: C/D vertical ratio; C/D H-ratio: C/D horizontal ratio; CCT: central corneal thickness; GCC: ganglion cell complex; GCC-FLV: GCC-focal loss volume; GCC-GLV: GCC-global loss volume; IOP: intraocular pressure; MD: main defect; RNFL: retinal nerve fiber layer thickness;

Table S2. Details of clinical correlation for DEMs in the aqueous humor of POAG patients.

Table S3. Details of clinical correlation for DEMs in the plasma of POAG patients.

Table S4. Purine metabolites correlation between aqueous humor and plasma.

|  | Correlation‡ | | Aqueous humor† | | | Plasma† | | |
| --- | --- | --- | --- | --- | --- | --- | --- | --- |
|  | r | p value | VIP | log_2_(FC) | q value | VIP | log_2_(FC) | q value |
| Urea | 0.75 | <0.001 | 0.66 | -0.079 | 0.49 | 0.46 | -0.13 | 0.41 |
| L-Glutamine | 0.43 | 0.048 | 1.3 | 0.22 | 0.012 | 0.43 | 0.11 | 0.37 |
| Cyclic Amp | 0.42 | 0.05 | 2.6 | -0.68 | <0.001 | 1.3 | -0.37 | 0.086 |
| 2,6-Dihydroxypurine | 0.4 | 0.063 | 1.2 | -0.25 | 0.025 | 1.5 | 0.96 | 0.059 |
| Uric Acid | -0.4 | 0.065 | 3.2 | 1.6 | 0.009 | 2 | -0.17 | 0.56 |
| Glycine | 0.29 | 0.2 | 0.38 | 0.082 | 0.56 | 0.48 | 0.18 | 0.37 |
| Adenosine | -0.14 | 0.55 | 0.3 | -0.039 | 0.37 | 1.9 | 4.9 | 0.11 |
| Inosine | 0.1 | 0.65 | 0.86 | -0.26 | 0.12 | 3.2 | 5.7 | 0.04 |
| Xanthosine | 0.081 | 0.72 | 1.9 | -0.65 | 0.03 | 0.41 | 0.021 | 0.3 |
| Adenine | 0.081 | 0.72 | 0.29 | -0.046 | 0.36 | 0.39 | -0.02 | 0.9 |
| Hypoxanthine | -0.037 | 0.87 | 2.1 | -0.75 | 0.006 | 2 | 2 | 0.04 |
| Guanosine | -0.032 | 0.89 | 0.89 | -0.26 | 0.15 | 2.2 | 5 | 0.17 |

‡: The correlation of each metabolite between aqueous humor and plasma was analyzed using Pearson correlation. †: Statistics of the metabolites in POAG group compared to control group.

Table S5. The influence of the administration of tropicamide before cataract surgery for DEMs in the aqueous humor

| DEMs | P value |
| --- | --- |
| Lysopa 16:0 | 0.01 |
| D-Sorbitol | 0.13 |
| Dulcitol | 0.24 |
| Hydroxyphenyllactic acid | 0.35 |
| 3'-Sialyllactose | 0.46 |
| PAF C-16 | 0.68 |
| Phenyllactate (Pla) | 0.68 |
| Lysopc 18:0 | 0.69 |
| Lysopc 16:0 | 0.69 |
| Lysopc 18:3 | 0.69 |
| Lysopc 18:1 | 0.70 |
| Lysopc 16:1 | 0.73 |
| L-3-Phenyllactic Acid | 0.75 |
| Barbituric acid | 0.76 |
| Lysopc 15:0 | 0.76 |
| N6-Succinyl Adenosine | 0.84 |
| Hypoxanthine | 0.86 |
| Hexadecanamide | 0.89 |
| Cyclic Amp | 0.91 |
| 2-Methylbenzoic acid | 0.94 |
| Xanthosine | 0.95 |
| Uric Acid | 1.00 |

Table S6. The overall drugs influence of the preoperative topical drugs on DEMs in POAG group.

| Aqueous humor | | Plasma | |
| --- | --- | --- | --- |
| Metabolites | P value | Metabolites | P value |
| Cyclic Amp | 0.79 | 3-(4-Hydroxyphenyl)-Propionic Acid | 0.7 |
| 2-Methylbenzoic acid | 0.74 | N-lactoyl-phenylalanine | 0.36 |
| 3'-Sialyllactose | 0.99 | 9-Hpode | 0.6 |
| Lysopc 18:0 | 0.26 | D-Mannitol | 0.69 |
| Dulcitol | 0.74 | Inosine | 0.92 |
| Lysopc 15:0 | 0.21 | Hypoxanthine | 0.94 |
| Hypoxanthine | 0.57 | Guanidinoethyl Sulfonate | 0.57 |
| Uric Acid | 0.043 | Hypoxanthine-9-β-D-Arabinofuranoside | 0.92 |
| Phenyllactate (Pla) | 0.021 | P-Aminobenzoate | 0.97 |
| Xanthosine | 0.67 | Hydroxyacetone | 0.55 |
| Lysopc 16:0 | 0.19 | 2-Aminoadipic Acid | 0.097 |
| Lysopc 18:3 | 0.22 |  |  |
| Hydroxyphenyllactic acid | 0.22 |  |  |
| Lysopa 16:0 | 0.65 |  |  |
| Lysopc 16:1 | 0.48 |  |  |
| Barbituric acid | 0.14 |  |  |
| L-3-Phenyllactic Acid | 0.15 |  |  |
| PAF C-16 | 0.22 |  |  |
| N6-Succinyl Adenosine | 0.047 |  |  |
| Hexadecanamide | 0.65 |  |  |
| Lysopc 18:1 | 0.3 |  |  |
| D-Sorbitol | 0.83 |  |  |


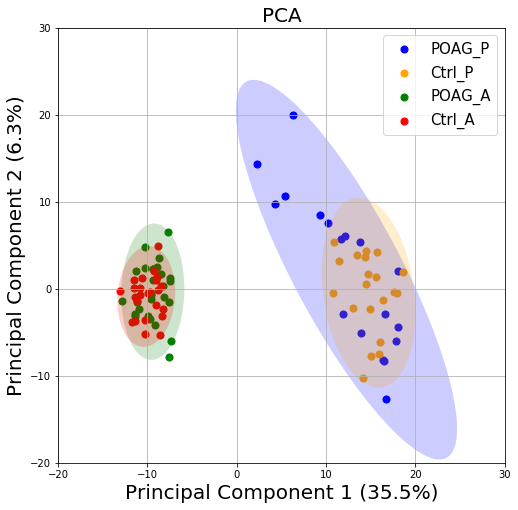


Figure S1. The PCA score plots of all samples.

Figure S2. The correlation matrix heat map of DEMs-A (A) and of DEMs-P (B).

Figure S3. Metabolite quantification. The total ion chromatography (TIC) chromatograms and MRM spectra obtained from the QC samples with (A, C) negative and (B, D) positive modes. Quantitative analysis integral calibration chart, the x-axis is the retention time (RT) of metabolite detection (min), the y-axis is the ion current intensity (cps) of a metabolite with (C) negative and (D) positive modes.

Figure S4. Quality control. The overlapped total ion chromatography (TIC) of the QC sample in (A) negative and (B) positive ion mode. The PCA score plots of all samples in (C) negative and (D) positive ion mode. (E) the coefficient of variation of 395 metabolite among all QC samples.


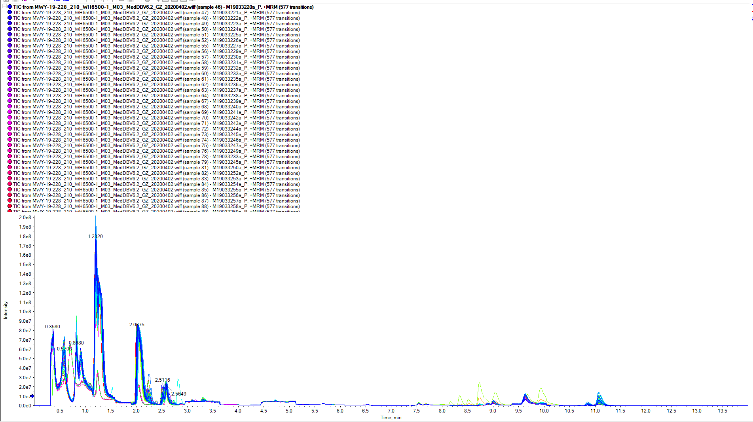


Figure S5. TIC_overlap chromatography data of aqueous humor samples


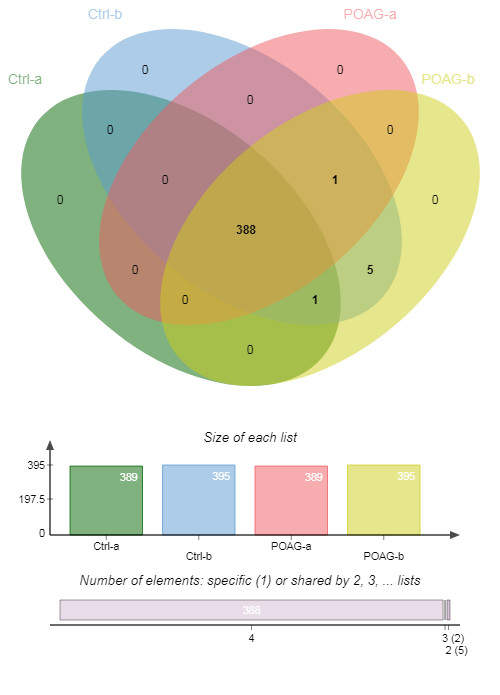


Figure S6. Venn diagram of metabolite numbers in groups.


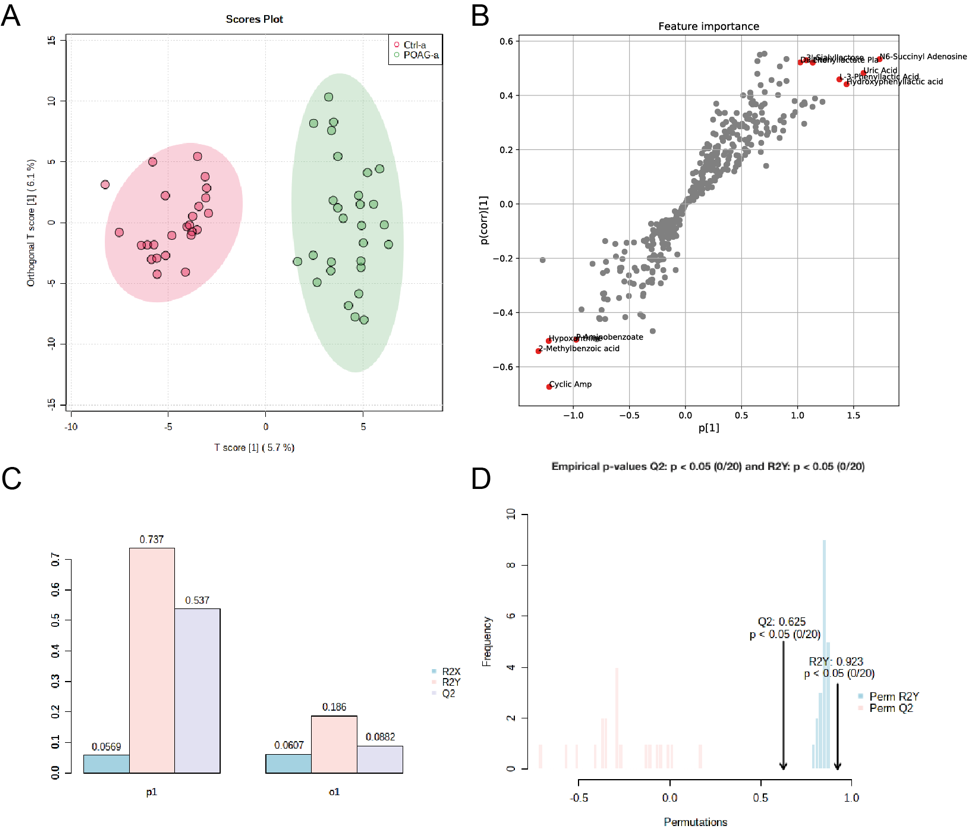


Figure S7. OPLS-DA results of aqueous humor samples in POAG and control groups. (A) The score plot. (B) The feature importance S-plot (p>0.95, p[corr]>0.4). (C) Model overview R^2^ = 0.737, Q^2^ = 0.537. (D) Perturbation result. DEMs-A discovered from OPLS-DA model were N6-succinyl adenosine, uric acid, hydroxyphenyllactic acid, L-3-phenyllactic acid, phenyllactate (Pla), 3'-sialyllactose, dulcitol, P-aminobenzoate, cyclic amp, hypoxanthine and 2-methylbenzoic acid.


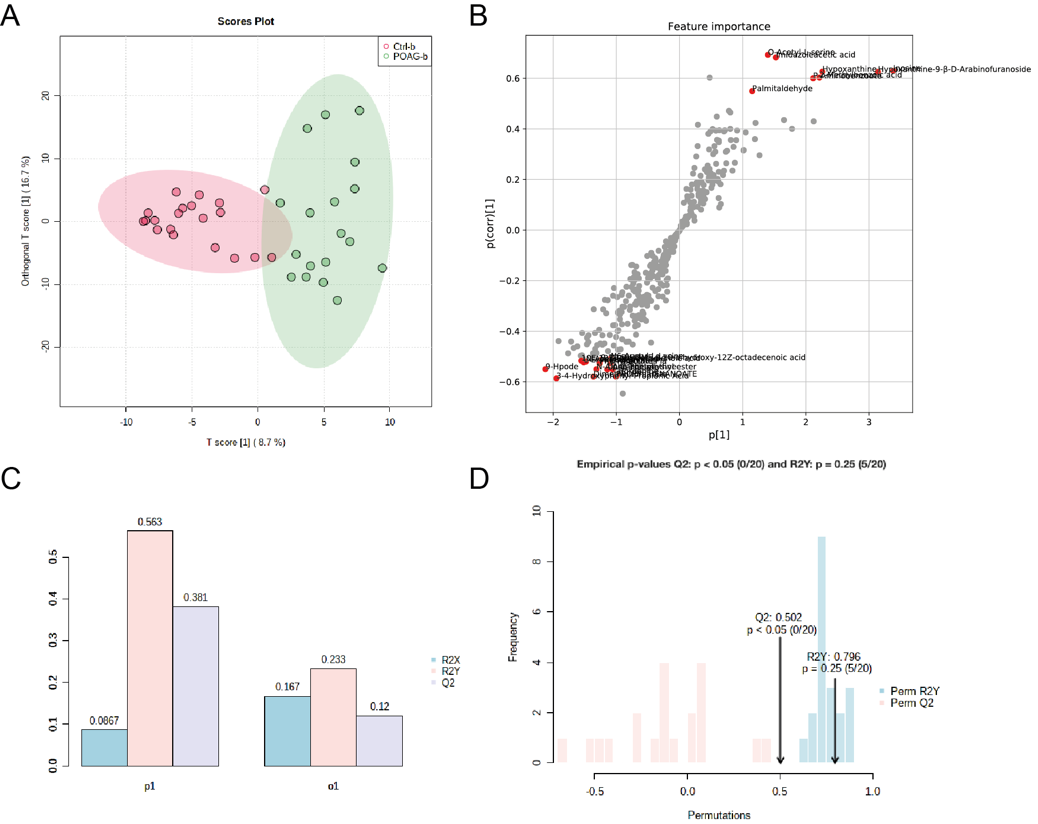


Figure S8. OPLS-DA results of plasma samples in POAG and control groups. (A) The score plot. (B) The feature importance S-plot (p>1, p[corr]>0.48). (C) Model overview R^2^ = 0.563, Q^2^ = 0.381. (D) Perturbation result. DEMs-P discovered from OPLS-DA model were P-aminobenzoate, inosine, hypoxanthine-9-β-D-arabinofuranoside, hypoxanthine, 9-hpode, 3-(4-hydroxyphenyl) propionic acid, N-lactoyl-phenylalanine and 2-Aminoadipic Acid.
